# Usability testing of a smoking cessation smartphone application (‘SmokeFree Baby’): A think-aloud study with pregnant smokers

**DOI:** 10.1177/2055207617704273

**Published:** 2017-04-12

**Authors:** Joyce Wu, Ildiko Tombor, Lion Shahab, Robert West

**Affiliations:** Department of Behavioural Science and Health, University College London, UK

**Keywords:** Smoking cessation, pregnancy, intervention development, usability testing, smartphone app, think-aloud, qualitative study

## Abstract

**Background:**

Only a few digital interventions have been developed for pregnant smokers, and little is known about the acceptability and usability of smartphone apps to aid cessation in pregnancy. This study aimed to explore pregnant smokers’ views on the design, content and usability of a pregnancy-specific smoking cessation app in order to inform intervention development and optimisation.

**Methods:**

Ten interviews were conducted and the ‘think-aloud’ protocol was used in order to explore participants’ views about a smoking cessation smartphone app (‘SmokeFree Baby’). The data were subsequently thematically analysed. Participants were 18 and over, pregnant, and daily or weekly cigarette smokers.

**Results:**

Three main themes were identified: views about the design elements, mode of delivery and content of the intervention. App design was considered as an important element that might influence potential users’ engagement with the intervention. Participants felt that the intervention content was educational, motivational and non-judgemental. However, it was emphasised that the app should provide further options for personalisation and include more practical features.

**Conclusions:**

Delivering smoking cessation support via a smartphone app can be feasible and acceptable for pregnant smokers. They appear to value content that is motivational, educational and personalised, and meeting these requirements may be important for user experience and promoting engagement with the intervention.

## Background

Stopping smoking at any stage of pregnancy has many health benefits, and mothers who successfully stop smoking by the third month of their pregnancy have similar risks for smoking-attributable pregnancy complications to those of non-smoking mothers.^1^ Effective smoking cessation support (e.g. behavioural support and nicotine replacement therapy (NRT)) is available for pregnant smokers within the National Health Service (NHS) in England,^2–4^ but it has low uptake due to a number of barriers relating to engagement with health professionals on a face-to-face basis (e.g. fear of being judged).^5^ Digital interventions may address some of these barriers, but little is known about their use in pregnancy.

Digital behaviour change interventions (e.g. web or mobile phone-based behavioural support) have become common in recent years,^6^ and some of these digital interventions that aid smoking cessation have been found to be effective in the general population.^7,8^ Only a few digital interventions have been developed for pregnant smokers,^9,10^ but studies have shown promising results in terms of feasibility and potential effectiveness.^11–16^ In order to advance the development of digital smoking cessation interventions for pregnant smokers, previous studies^11–16^ have called for future research to establish optimal methods of delivering theory-based interventions on different digital platforms, the level of personalisation required, the structure and regularity of intervention delivery, and the usability of the intervention in relation to its design and content elements. However, to the best of our knowledge, no study has been published on the usability evaluation of a smoking cessation aid for pregnant smokers using a smartphone app as a platform for intervention delivery.

The ‘SmokeFree Baby’ smartphone app (www.smokefreebaby.co.uk) has been developed to provide a readily available smoking cessation aid for pregnant smokers free of charge.^10^ The intervention development was informed by the Multiphase Optimisation Strategy,^17^ the UK Medical Research Council guidance,^18^ the Behaviour Change Wheel,^19^ the COM-B model of behavior,^20^ the plans, responses, impulses, motives, evaluations (PRIME) theory of motivation,^21^ evidence from the scientific literature, and 42 behavioural change techniques (BCTs) from the BCT Taxonomy v1.^22^ The app is divided into a general app feature (‘Toolbox’) and five experimental modules (‘Identity’, ‘Stress Relief’, ‘Health Effects’, ‘Face-to-Face’ and ‘Behaviour’) in order to evaluate their effects in a factorial experiment. Each experimental module has an intensive (with interactive features) and minimal (text-only) version, and a key intervention target, as follows. The ‘Identity’ module provides advice to help pregnant women establish a new non-smoker identity, the ‘Stress relief’ module addresses stress management, the ‘Health Effects’ module provides information about the health effects of smoking and benefits of cessation, the ‘Face-to-Face’ module provides ready access to stop smoking services in the localities, and the ‘Behaviour’ module provides distraction from urges to smoke. The ‘Toolbox’ feature provides information about a range of topics, including NRT use, social support and the amount of money that pregnant women have saved by not smoking.

Developing complex digital interventions requires an iterative process of evaluation and refinement of the intervention. An essential step in this process is to conduct usability testing in order to explore potential users’ views about the intervention and evaluate its acceptability and feasibility in the target population.^23^ Previous studies have reported the usability evaluation of various internet-delivered health care interventions^24,25^ and smartphone apps, such as to improve self-management strategies among young adults with sickle cell disease^26^ and improve self-management of pain among people with chronic or recurrent pain episodes.^27^ A common method for usability evaluation of health behaviour interventions is the think-aloud method.^25^ In order to inform future development of digital interventions, particularly mobile technologies, this study aimed to explore pregnant smokers’ views on the design, content and usability of a smoking cessation app by applying the think-aloud method.

## Methods

### Design

The think-aloud method was used to conduct the interviews. In order to gain insights into participants’ cognitive process and attitudes towards the SmokeFree Baby application, this protocol involved asking participants to freely verbalise their thoughts and feelings they might have whilst engaging with the intervention.^23,25^ The SmokeFree Baby app was downloaded from the Apple App Store by the interviewer onto a smartphone, which was provided for participants during the interviews. All interviews were audio-recorded, and the screen of the smartphone was video-recorded in order to document which sections of the app participants were using during the interviews. Participants received high-street vouchers worth £30 to compensate them for their time and effort. Ethical approval was obtained from the University College London’s Psychology and Language Sciences Departmental Ethics Committee (Project ID: CEHP/2013/508).

### Participants

Advertisements for the study were distributed to charities and stop-smoking clinics in London, and online advertisements were placed on pregnancy-specific forums and local community websites. In order to take part, participants had to be at least 18 years of age, be pregnant, and smoke cigarettes at least once a week. Twenty-six people responded to the recruitment advertisement, and ten pregnant smokers were interviewed. Sixteen individuals were excluded from taking part in the study, as they either stopped corresponding, were not able to participate due to travel, or did not meet the eligibility criteria. Those under the age of 18 who did not smoke at least once a week and were no longer pregnant were excluded. All participants were naïve users, as they had not engaged with the SmokeFree Baby app prior to the interview.

### Procedure

Informed consent was obtained from all participants prior to the interviews. Participants completed a brief questionnaire asking them about their age, weeks of pregnancy, education, motivation to stop smoking (‘How motivated are you to give up smoking at this attempt?’, ranging from 0 ‘Not motivated at all’ to 3 ‘Extremely motivated’), as well as confidence in their ability to quit (‘How confident are you in your ability to stop smoking?’, ranging from 0 ‘Not confident at all’ to 3 ‘Extremely confident’). Nicotine dependence was measured using the Heaviness of Smoking Index.^28^ Data from the background questionnaire were used for contextual understanding of the study.

The complete test version of the app was used, in which the intensive version of each experimental module was presented to participants, in order to evaluate all app features. There were no practice trials, but the interviewer explained the think-aloud method through a brief example to ensure that participants understood the method. Participants were asked to say out loud anything that came across their minds whilst using the app, and they were encouraged to make both positive and negative comments. Participants were also informed that the interviewer was not involved in the development of the app. If a participant stopped talking, the interviewer intervened by prompting her with open-ended questions, such as ‘What are your thoughts right now?’ to maintain the flow of conversation.

There was no time limit to the interviews, and there were no restrictions on the length of response a participant could give. Before finishing the interview, the interviewer prompted participants to revisit features that had not been explored and encouraged them to say out loud what they thought about those sections. Each interview lasted approximately 30 minutes.

### Data analysis

Interviews were transcribed verbatim and each participant was assigned a code for identification. The data were analysed thematically.^29^ Transcripts were read and re-read by JW to familiarise herself with the dataset. An initial coding framework was generated manually around emergent themes. Recurrent themes and subthemes were identified in an iterative process by JW and IT, which involved checking the coding for consistency and developing and refining the thematic framework. Illustrative quotes for each theme were selected. No analytic software was used for data analysis.

## Results

Table 1 reports participants’ socio-demographic characteristics. In the thematic analysis, three main themes were identified in participants’ accounts: views about the design elements (e.g. aesthetics of the app), mode of delivery (e.g. functionalities in the app) and intervention content (e.g. usefulness). Each theme and related subthemes with illustrative quotes are described in detail below, and further quotes are reported in the supplementary file (Table S1).

**Table 1.** Participants’ demographic and background characteristics.

	Total (*n* = 10)
Age, mean (SD); range	28.5 (5.7); 19–36
Weeks of pregnancy, mean (SD); range	12.9 (7.3); 8–29
Highest level of education completed, % (*n*)	
High school	50.0 (5)
Bachelor degree	40.0 (4)
Masters or above	10.0 (1)
Heaviness of Smoking Index, mean (SD)	1.5 (1.9)
Motivation to give up smoking at this attempt, mean (SD)	2.2 (1.1)
Confidence in ability to stop smoking, mean (SD)	1.2 (0.9)

### I. Pregnant smokers’ views about the design elements

#### 1. Aesthetics

Generally, pregnant smokers felt that the app was well designed. Although the colour, font type and visuals in the app were perceived as appealing, participants noted that the overall user experience could be improved by using a variety of colours to highlight different features, adding an option for customisable colours, increasing the font size, and adding animated elements to various app features.

It would be nice if it was a bit alive, like animated text, I don’t know. I’d quite like that. You scroll and it kind of just flows. I mean there is no difference between reading a book and reading this. So I guess I want to look at something quite lush visually. (P6, 33 years old)

#### 2. Navigation

**Figure 1.**
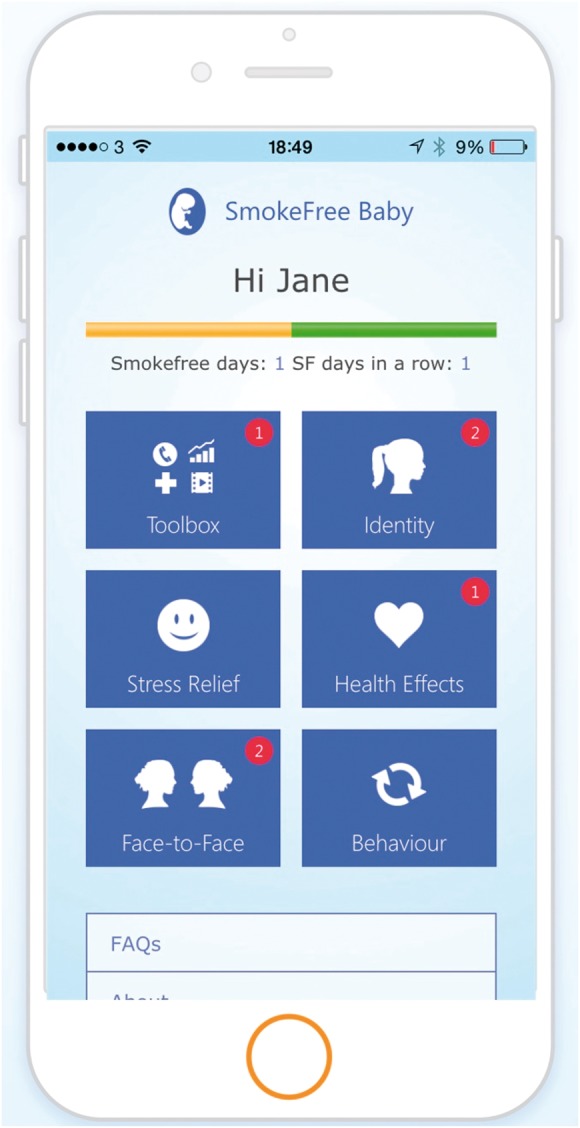
Screenshot of the upper half of the main dashboard with easy-to-discover features (Toolbox, Identity, Stress Relief, Health Effects, Face-to-Face, Behaviour) that drew participants’ attention naturally.

**Figure 2.**
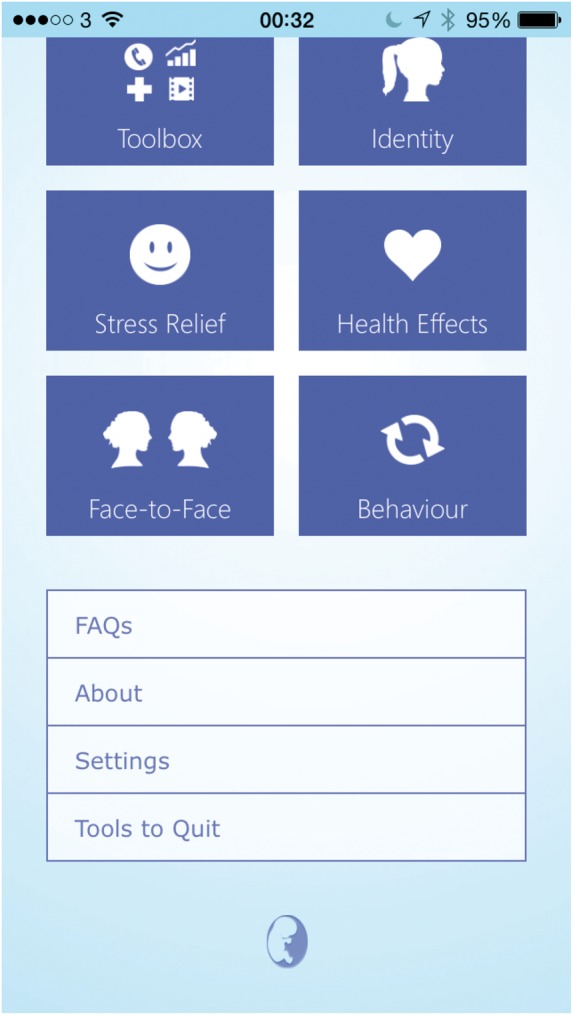
Screenshot of the bottom half of the main dashboard with less prominent features (Frequently Asked Questions, About, Settings, Tools to Quit) that often remained undiscovered without prompting.

Participants reported that it was easy to operate and navigate between different parts of the app. Of the main features, some participants were naturally drawn to the ‘Stress Relief’ feature upon entering the application (Figure 1). However, a number of potentially useful features that were less prominent in the design, such as ‘Frequently Asked Questions’, ‘About’, ‘Settings’ and ‘Tools to Quit’, would sometimes remain undiscovered by users without prompting (Figure 2).

I wouldn’t have found that [‘Tools to Quit’ feature]. That’s good because it tells you what you need to do to quit, so I quite like that. Maybe it should actually be at the top somewhere, because by doing that you know where you need to go instead of doing it at the end, which is quite pointless. (P8, 19 years old)

The importance of presenting the intervention content, such as the tips to help women cope with stress, in shorter segments instead of a long list of tips was also emphasised.

It [a feature within stress management tips] is quite helpful, but I think the layout, because this is at the top and sometimes I wouldn’t even think to scroll down in a way. (P9, 26 years old)

### II. Mode of delivery of intervention content

#### 3. Functionality

Easy access to intervention content, such as contact details of local stop smoking services, both online and offline, was perceived as important.

You would find them [contact details of stop smoking services] on Google, but having a number there at hand that you can use straight away is always helpful. (P4, 25 years old)

Participants commented that the app felt interactive and provided lots of functionality by means of different app features in which the content was delivered in various ways (e.g. quizzes, videos) going beyond a more traditional text-based format.

I quite like the quiz. It’s very interactive, which is really good and it helps you learn more about what you are actually doing to your body and to your baby’s body without actually doing it in a patronising way. (P8, 19 years old)

It was also noted that the app could include a pedometer feature to facilitate physical activity during the quit attempt, and pregnant smokers’ reviews of different NRT products in order to help others decide which products they should try.

I think what they can add to that [a feature about smoking cessation medications] is they could say people can rate them [different NRT products] as to which ones are popular and which brands are good ones. (P3, 31 years old)

Although some app features were perceived as unnecessary (e.g. adding contact details of friends to have ready access to social support), most women found it useful to make use of the built-in functions of their smartphones (e.g. camera) as part of the intervention.

That’s [a video diary feature] helpful, because you can take pictures of your skin, you can take pictures of your teeth, you can take pictures and videos of your changes and your body and that’s helpful. (P7, 20 years old)

#### 4. Regular update of content

In order to motivate users to maintain engagement with the intervention, pregnant smokers argued that the content of various app features should be updated on a daily basis. Therefore, the daily tips and videos were seen as potentially useful, but it was also emphasised that the app could be improved by including more videos from a number of different pregnant ex-smokers.

This [a ‘Tip of the Day’ feature] would be helpful, as long as it is everyday. If it comes back say four days in with the same thing, then that’s going to annoy me, because I feel like I have just wasted my time, so it’s got to be different everyday, and that’s seven days a week really. (P1, 30 years old)

#### 5. Availability of help

Pregnant smokers emphasised that it was important for them to know that help is readily available and non-judgemental expert advice is always at hand through the app when they need it. From their perspectives, videos with stop smoking advisors were particularly useful, as these were encouraging and provided good-quality smoking cessation advice similar to the experience of visiting a smoking cessation clinic.

I feel like I have gone to the smoking cessation nurse without going to the smoking cessation nurse. (P4, 25 years old)

#### 6. Language

Pregnant smokers felt that the tone of the app was not patronising or intimidating, and the difficulties of giving up smoking during pregnancy were recognised. Therefore, they would recommend SmokeFree Baby to their friends, as advice to quit was communicated in a non-judgemental way.

I like the fact that they’ve recognized that [it can be difficult to stop smoking] because to be honest, when it comes down to your general doctor, they are not very understanding. Most of them haven’t smoked, so they don’t really know how hard it actually is. But the fact that this app here just that little bit there recognises that it is something that is not going to happen overnight, it’s quite reassuring. (P1, 30 years old)

On the other hand, many participants said that the language used to deliver specific aspects of the intervention content (e.g. health effects of smoking) was too technical and therefore would discourage women from maintaining engagement with the app.

I think the issue that I have with this is when it gets too technical. It’s too much, it’s too much thinking, I don’t want to deal with it. It just needs to be quick and straight to the point and get the message across. (P3, 31 years old)

### III. Pregnant smokers’ views about the intervention content

#### 7. Usefulness

Generally, participants perceived the intervention content as educational and informative. They felt that the app covered a range of topics, some of which were seen as particularly important (e.g. stress management tips), provided detailed information about smoking cessation, and included a sufficient amount of tips and advice to help women with their quit attempts during pregnancy.

There is a lot to play around with there that pretty much covered all grounds, I would say. I can’t think of anything else you could possibly put on here really. (P4, 25 years old)

Participants emphasised that the app provided educational information they did not know previously, especially in relation to the health effects of smoking during pregnancy, NRT and smoking cessation medications, and the different types of face-to-face support available for pregnant smokers.

The other options that you have: hours, home visit, which I was not aware of. Telephone support is good. The fact that you can drop into any clinics in your area, I didn’t actually know that either. It’s given me extra information so far. (P1, 30 years old)

Some women mentioned that the app content was mainly knowledge-based and commented that more practical features would be needed in addition to the breathing exercise or distraction game, even though the usefulness of these features was sometimes questioned.

Giving me the theory of why I shouldn’t smoke and what is the experiences of people I don’t know. How does that help me? I don’t feel that helps me stop. Like with the distraction game, that’s something that is really there to help me at the moment when I need it. (P3, 31 years old)

#### 8. Personal relevance

Most women in this study reported that they were able to relate to the content of the app on a personal level, and they found it particularly inspiring to watch videos with a pregnant ex-smoker talking about her experiences with quitting.

I can imagine this would help quite a lot: talking to ex-smokers about what helped them quit and then all different sections [in the ‘Identity’ module]. This is really good, because I think you need communication with other people who have gone through the same situation as you to make you feel like you are not alone. So this is probably my favourite section so far. (P8, 19 years old)

However, some participants recognised that a number of tips would not be relevant to them due to their individual motivational background or life circumstances, and felt that the content was not personalised enough to meet their individual needs, as it did not provide a structured quit plan tailored to their cigarette dependence.

Something more personalised just for you. It needs to be personalised definitely. It can’t just do it all for everyone […] because some are heavy smokers and some are light smokers. (P10, 29 years old)

#### 9. Motivational properties

Pregnant smokers felt that various aspects of the app (e.g. health quiz, video diary of their progress) were thought-provoking and boosted their motivation to stop smoking, especially when the content prompted them to think about their baby.

That makes you think more about your situation and that you are planning to have a baby, and it motivates you more. (P5, 36 years old)

However, some participants expressed that it is difficult to remain motivated in some situations even with help from the app available at hand, and that using distraction and motivational support may not always prevent lapses or relapse.

Once the seed is planted you want a cigarette, you would just look forward to the moment till the end of this [distraction features] to have a cigarette, because that’s what happens. Because if I want a cigarette, I would be like okay, I am going to do that [distraction game] and that [distraction quiz] and I am going to reward myself with a cigarette. (P3, 31 years old)

Participants noted that monitoring the number of smoke-free days could help them maintain their motivation to remain abstinent; however, monitoring the amount of money saved by not smoking could be both motivational and potentially annoying.

That’s [progress bar] quite motivational, because I guess if you have gone so far, you don’t want to ruin it. (P8, 19 years old)

I don’t want it to tell me how much I’ve saved, because I remind myself how much I have saved by having extra money available. For me, it does come across as a bit pushy by telling me ‘oh you saved this amount of money’. I’ll be like ‘yeah … what is your point’. I will know that, you don’t need to tell me. (P1, 30 years old)

Finally, participants emphasised that it could be motivational to learn more about how pregnant ex-smokers changed their habits during the quit attempts and how they coped with cravings and withdrawal.

I think it would be very helpful if there were videos of her […] showing her daily routine, saying ‘I really feel like I need a cigarette right now guys, and this is what I am going to do.’ […] So because I feel like if she is real, I can observe her resist her cravings, then I will feel like I can learn something, like even from her emotions or something. (P6, 33 years old)

## Discussion

This study provides in-depth insights into users’ experiences with SmokeFree Baby in order to better understand how smoking cessation smartphone apps should be designed and configured to meet the needs of pregnant smokers. Findings of this study suggest that apps need to visually appeal to this specific target group, as the design of the app may influence potential users’ willingness to engage with the intervention. Delivering smoking cessation support in a non-intimidating and non-patronising manner and through interactive features seems highly valued by pregnant smokers. Participants found the motivational and informative properties (e.g. evidence-based information regarding the effects of smoking on their health) of the intervention content important, but thought the text should be easy to understand. Providing fresh content on a regular basis, particularly in relation to cravings management, appears to be an essential requirement to maintain the engagement of pregnant smokers with the intervention. Findings from this study also indicate that social support and personal relevance in relation to the content and personalisation of the app are important for this target population.

In line with previous findings regarding text-message programmes,^15^ this study found that availability of help and having non-judgmental advice at hand were seen as important aspects of a smartphone app to aid cessation during pregnancy. Receiving educational content in relation to smoking and cessation was also perceived positively by pregnant smokers.^13^ Similarly to internet-based smoking cessation interventions,^12^ having regular content updates, practical features and a variety of features could be desirable qualities of pregnancy-specific smoking cessation apps. Although information and practical features in relation to cravings and withdrawal are provided in SmokeFree Baby, it appears that pregnant smokers would have preferred additional tools to cope with cravings. Since pregnant smokers valued the social support component of the app, future intervention development may take advantage of smartphone technology as a communication tool and elevate the types of social support available in apps (e.g. provide a platform for users to share real-time advice and experiences with each other). Although this qualitative study supports the importance of providing personalised content in digital smoking cessation interventions in pregnancy,^12,14^ as it was perceived to be more engaging than non-personalised features, quantitative indices of engagement with the intervention (e.g. number of logins to the app) would need to be evaluated.

A limitation of this study is that it only evaluated the SmokeFree Baby application, and although it provides potentially useful inputs for the development of future digital interventions for pregnant smokers, the results may not be generalisable to all types of digital aids. Another limitation is the relatively small sample size, as despite using multiple channels for recruitment for almost a year, only 10 participants were interviewed. However, this is in line with previous studies,^11,12^ which also found it difficult to engage pregnant smokers to participate in research studies.^14,30,31^ For example, similarly to this study, fewer than 40% of pregnant smokers who were invited were willing to take part in a telephone interview to explore their views about internet-based interventions for smoking cessation,^12^ even though participants did not need to travel to meet with the researcher in person, unlike pregnant smokers in this study. Moreover, guidelines^32^ have suggested that approximately 6–10 interviews can be considered appropriate for a small qualitative study in order to conduct in-depth analysis from the material collected. The development and testing of digital interventions needs to be done iteratively, and the aim of this study was to recruit a sample that was feasible within the time and research constraints, and also sufficient to inform the refinement of the intervention. Although participants received a thorough briefing on the think-aloud protocol at the beginning of the interview, they sometimes tried to engage with the interviewer, and consequent input from the interviewer may have influenced participants’ cognitive processes.^33^ The interviewer only provided inputs when it was absolutely necessary, and her involvement was non-directive in order to minimise bias. Lastly, as is the case for all qualitative research, the study does not allow generalisation beyond the immediate sample. However, this type of qualitative analysis has been recognised as a useful and essential step to evaluate the usability of digital intervention tools as part of their stages of development.^34^

This study contributes to a better understanding of pregnant smokers’ views about digital smoking cessation interventions, which is a relatively new, emerging field in the literature. It appears that a smartphone app can be a suitable medium to provide expert advice and social support for pregnant smokers and deliver smoking cessation intervention during pregnancy. While the effectiveness of smoking cessation apps in pregnancy has yet to be investigated, and quantitative usage data will need to validate these findings, this study suggests that the SmokeFree Baby app is acceptable and potentially useful for its target population.

## Supplementary Material

Supplementary material

## Supplemental Material

The online supplementary material is available at http://journals.sagepub.com/doi/suppl/10.1177/2055207617704273.
