# Supplementary material for: Usability testing of a smoking cessation smartphone application (‘SmokeFree Baby’): A think-aloud study with pregnant smokers
Source: Digit Health. 2017 Apr 12;3:2055207617704273. doi: 10.1177/2055207617704273 (PMC6001180; doi:10.1177/2055207617704273)
Supplement: Supplementary material [file DHJ704273_supplementary_material.pdf]

Table S1. List of main points identified in each sub-theme with illustrative quotations

	Main Findings	Quotations
**I. Pregnant smokers' views about the design elements**<br><br>**1.Aesthetics**		
**1.1. Colours**:	The app could be improved by using a variety of colours to highlight features	"It's very blue isn't it. […] maybe if you get to pick what colour it is as a customisation thing that would be quite good." *(P8, 19 years old)*<br>"I like the fact that it is blue, but I don't think everything should be blue. Some needs to have certain bits of it highlighted. For instance if you kept 'Identity' [module] and 'Toolbox' as blue that would be great. But with 'Stress relief' [module] and 'Health Effects' [module] say like in a red colour, and may be a different colour for 'Face to face' [module] and 'Behaviour' [module]. That way it highlights it a bit more; different aspects of what actually available."*(P1, 30 years old)*
**1.2. Font size and font type**	The font size should be increased in the future	"That's quite nice that you have little things about what it is…how it's helpful …it is quite small [The font]." *(P9, 26 years old)*
**1.3. Visuals**	The app was generally considered to be visually appealing	"It's a really nice layout I like the colours…I love the little pretty writing…to be honest, it's quite nice." *(P6, 33 years old)*

**2.Navigation**		
**2.1. Operation of app features**	It was easy to operate and navigate between components	"Every time I go on the home page, it shows how much I can save and different things I can buy which is useful...everything that is off the home screen is also available on the screen at the bottom as well, that could be good." *(P4, 25 years old)*
**2.2. Visibility of potentially useful features**	Some potentially useful features were less prominent	"This is really good; you should put this really big so you can pay attention to it at the start." (*P9, 26 years old)*
**2.3. Presenting the intervention content in shorter segments**	Contents should be given in shorter segments	" I don't think it's [tips of the day] too much, I think if they were all that long then maybe might struggle." *(P7, 20 years old)*<br>"Maybe for a tip it's a little too much, it's good what they are saying, maybe they can rephrase it in a simpler way." (*P8, 19 years old)*"It's [Breathing Exercise feature] really clever actually, it's not too long, you know if it was too long you would get stressed again thinking this isn't working but it just shows you enough to calm you down for that minute." *(P4, 25 years old)*

**II. Mode of delivery of intervention content**<br><br>**3. Functionality**		
**3.1. Easy access to intervention content**	Useful information are available to users both on and offline	**“**So instead to having to call a helpline or anything like that, I have it on my phone where it just a personal thing that would make me feel a lot more stress free.” *(P1, 30 years old)*
**3.2. Interactive content**	Intervention content is readily available	*“*My favourite thing is the health quiz. Maybe it would be good if they can do some sort of game or interactive thing with these two parts [‘My Baby’ and ‘My Body’ features] as well.” *(P8, 19 years old)*
**3.3. Using the built-in functions**	The app utilised smartphone functionalities e.g. camera	“Would be nice to document a pregnancy [using buit-in camera on smartphone] with the ‘Video Diary’ function but not sure if I would want to document it from a smoking point of view. I would probably use it to say: “okay, this me and I am that far gone and I am not smoking anymore, I am cutting down” so I can confirm it in myself that’s who I am: a non smoker now.” *(P4, 25 years old)*

**4. Regular update of content**		
**4.1. Daily updates**	It is important to update different types of content on a regular basis to maintain motivation and interest for users	**"**It's ['Distraction Q&A' feature] a nice idea but then you get very bored of these two games very fast. I think there maybe needs to be something else, but I don't know because you then have to keep updating it." *(P8, 19 years old)*
**4.2. Different video contents**	It would be beneficial to have different speakers in the videos for variety and also to learn about different perspectives and experiences	"I like that I definitely felt the women's [Women from the 'ex-smokers videos' feature] urge and it's really good to hear someone who have gone through it, it would be good to have a few different people so it shows that more people have achieved it as well." (*P9, 26 years old)* "If someone has gone through all the videos, then they have no more use for the app…maybe what would be more helpful is to update the app, once a week with new videos…so it makes you want to go on the app again." (*P7, 20 years old)*
**5. Availability of help**	Users felt that it was important to know that help and expert advice is readily available.	"She [Midwife from 'Pro Advice' feature] makes you feel like there is someone there helping you rather then just have to read it to motivate yourself. Sometimes it's helpful to have someone speaking." *(P9, 26 years old)*

**6. Language**		
**6.1. Non-judgemental tone**	The tone of the app was perceived as non patronising and non intimidating	“She [Advisor from ‘Pro advice’ feature] is not intimidating, she is like your everyday women who you would sit down and you can be completely honest with her, I like that.” *(P3, 31 years old)* “Having someone [Advisor from ‘Pro advice’ feature] inspirational and non judgmental…because to even use the app I need to feel like I am alright and I am not a terrible person...this feels like real human being support even if it’s a recording.” *(P6, 33 years old)*
**6.2. Technical language**	Technical language may discourage users to use to continue using app	“Bronchiolotis and fla […] these words are so big.” *(P5, 36 years old)*

**III. Pregnant smoker's views about the intervention content**		
**7. Usefulness**		
**7.1. Educational and informative**	The content was generally perceived as educational and helpful	"It's very informative. All these are eye-openers. I like the 'My Baby' and the 'My Body' [feature]. It is really important, because it goes into detail about everything." *(P2, 36 years old)*<br>"They [Questions from 'Health Quiz' feature] are thought provoking…I have learnt something." *(P4, 25 years old)*
**7.2. Novel information to users**	Users were able to learn new information about smoking and pregnancy during their time exploring the app	"[Reading from 'My Body' [module] Damage soft tissues…wow its really good to know these things, I probably shouldn't smoke that much." *(P6, 33 years old)*
**7.3. Inclusion of additional practical features**	Practical features should be revised to help users cope with cravings during their moment of weakness	"It would be great if in 'Stress Plan' [feature] [there were] some practical coping strategies that are quick and easy than pulling a cigarette." *(P4, 25 years old)*
**8. Personal relevance**		
**8.1. Personally relevant and inspirational content**	The app content was relatable on a personal level	"I would listen to it [Woman from 'ex-smoker' feature] over and over again, just try to grasp it from her point of view, how she's done that, it

		would be more inspirational for me to say, okay well if this lady feels this way then that's something I want to feel and the route I want to go down." *(P1, 30 years old)*
**8.2. Not enough personalisation**	Advice and tips may need to be further personalised to suit individual differences	"Motivation to stop differs so greatly from one person to other. To think it's so difficult to hear someone's story and think that something will kick into you, it won't. You need your own motivations, something really need to click to you that is really personal to you, your personal reason." *(P3, 31 years old)*
**9. Motivational properties**		
**9.1. Motivational content**	Various contents were encouraging and provided motivation for users to think about their end goal	[After completing the 'Health quiz' feature] Six out of ten, hopefully I should be able to get all of those correct and be completely clued up and not be smoking. *(P4, 25 years old)*
**9.2. Limitations of the motivational effect**	Some distractions may not necessarily have enough effect to discourage users from ultimately smoking	"My guess in a sense yes it [distraction tip] can distract you for a little while, but it's not going to last forever." *(P1, 30 years old)*
**9.3. Monitoring of progress**	Monitoring of progress enables users to get an overview at a glance from display	"It's quite good because you never really realise how much [money] you are losing until you add it all up, its quite a shock so it's quite nice." *(P8, 19 years old)*<br>It ['Toolbox' feature] shows me how much I can save which is good…always an issue when you find out you are pregnant." *(P4, 25*

		years old)
**9.4. Additional motivational content**	The app was able to incorporate encouragement from users' social group	[Memo feature] "Help maintain our motivation to stop smoking by recording supportive video messages from your friend and family…that's a really good one, I would do it for myself or get my partner or my mum to do that for me." *(P2, 36 years old)*
